# Clinical characteristics and quality of life in Chinese patients with multiple system atrophy

**DOI:** 10.1002/brb3.1135

**Published:** 2018-10-30

**Authors:** Juan‐Juan Du, Tian Wang, Pei Huang, Shishuang Cui, Chao Gao, Yiqi Lin, Rao Fu, Haiyan Zhou, Shengdi Chen

**Affiliations:** ^1^ Department of Neurology & The Collaborative Innovation Center for Brain Science Ruijin Hospital affiliated to Shanghai Jiao Tong University School of Medicine Shanghai China; ^2^ Co‐innovation Center of Neuroregeneration Nantong University Nantong China

**Keywords:** health‐related quality of life, multiple system atrophy, Parkinson’s disease

## Abstract

**Objectives:**

Multiple system atrophy (MSA) is a progressive neurodegenerative disorder that causes early sustained disability and poor health‐related quality of life (HrQoL). The clinical features and their effects on the HrQoL of patients in China have received little attention in the research literature. We evaluated the clinical characteristics and HrQoL of Chinese patients with MSA.

**Materials and Methods:**

A total of 143 patients with MSA from the Department of Neurology, Shanghai Ruijin Hospital, were enrolled in the study from March 2014 to May 2017. Basic demographic data, motor symptoms, non‐motor symptoms, and HrQoL were assessed and compared with data from 198 patients with idiopathic Parkinson's disease (PD) who were matched by age, gender, and disease duration. Factors influencing the HrQoL of MSA patients were also analyzed.

**Results:**

The ratio of patients with predominant parkinsonism (MSA‐P) and prominent cerebellar ataxia (MSA‐C) was 95:48 among the 143 MSA patients. MSA‐P patients had a longer disease duration (*p* = 0.002), higher levodopa equivalent daily dose (*p* < 0.001), higher scores on the Unified Multiple System Atrophy Rating Scale (UMSARS) I (*p* = 0.026), UMSARS II (*p* < 0.001), UMSARS IV (*p* = 0.019), the Hamilton Rating Scale for Depression (*p* = 0.001), the Hamilton Anxiety Scale (*p* = 0.013), and lower scores on measures of olfaction (*p* = 0.021) and cognitive function (*p* = 0.044) than the MSA‐C patients. Stepwise regression analysis showed that depression, anxiety, degree of disability, and disease severity were independent predictors of decreased HrQoL.

**Conclusions:**

The results indicate that MSA‐P patients have more severe motor impairment, hyposmia, depression, anxiety, cognitive impairment, and lower HrQoL than MSA‐C patients. Depression, anxiety, degree of disability, and disease severity are predictors of poor HrQoL among Chinese patients with MSA.

## 1 INTRODUCTION

Multiple system atrophy (MSA) is a progressive neurodegenerative disorder characterized by a cluster of symptoms, including parkinsonism, cerebellar ataxia, pyramidal damage, and autonomic dysfunction, and the prevalence of MSA is 3–5/100,000 in the general population (Gilman et al., 2008). The two main subtypes of MSA are MSA with predominant parkinsonism (MSA‐P) and MSA with prominent cerebellar ataxia (MSA‐C). The etiology of MSA is unknown, although the formation of glial cytoplasmic inclusions (GCIs) and selected neuronal loss is thought to have some relationship with MSA (Ubhi, Low, & Masliah, 2011). Similar to idiopathic Parkinson’s disease (PD), a group of non‐motor symptoms consisting of autonomic dysfunction, rapid eye movement sleep behavioral disorder (RBD), depression, and anxiety, are common and severe in patients with MSA (Ghorayeb et al., 2002; Kirchhof, Apostolidis, Mathias, & Fowler, 2003; Palma et al., 2015; Schrag et al., 2010). Differences between PD and MSA have been investigated in recent years; for example, one study reported impaired olfaction in patients with PD, and relatively intact olfaction in those with MSA (Izawa, Miwa, Kajimoto, & Kondo, 2012). However, few studies in China have performed overall comparisons of patients with MSA and those with PD.

A cohort study revealed that MSA caused early sustained disability and poor health‐related quality of life (HrQoL), with a median survival of 9.8 years from symptom onset (Low et al., 2015). Considering the short survival and treatment difficulties associated with MSA, it is important to improve the HrQoL of the patients as soon as possible. The few studies that have evaluated the HrQoL of patients with MSA (Schrag et al., 2006, 2010; Zhang et al., 2017) have reported that depression and disease severity were the most common determinants of HrQoL (Schrag et al., 2006, 2010). Although the measure of quality of life in MSA patients has been conducted in western countries, there is only one study that assessed the HrQoL of Chinese MSA patients by the Parkinson’s Disease Questionnaire (PDQ‐39) (Zhang et al., 2017); the non‐motor symptoms were only evaluated using a non‐motor symptoms scale (NMSS) in that study, and PDQ‐39, which is designed specifically for use with PD patients, has limited validity when used with a sample of MSA patients (Schrag, Jenkinson, Selai, Mathias, & Quinn, 2007).

A comprehensive and detailed evaluation of Chinese patients with MSA is lacking. Our study focused on differences in clinical features between MSA and PD, and the determinants of poor HrQoL among patients with MSA using a battery of neurological assessment scales.

## 2 PATIENTS AND METHODS

### 2.1 Subjects

We recruited 143 Chinese patients with MSA from the movement disorder clinic and ward at Department of Neurology, Ruijin Hospital affiliated to Shanghai Jiao Tong University School of Medicine from March 2014 to May 2017. All the patients were diagnosed with probable or possible MSA, according to the second consensus statement on the diagnosis of MSA by movement disorder specialists (Gilman et al., 2008). The exclusion criteria were as follows: disease duration over 10 years, onset under 30 or over 75 years old, history of a secondary neurological disease, such as epilepsy, brain tumor, head trauma, stroke, dementia, or cerebral small‐vessel disease, and evidence of a physical illness that could interfere with participation in a clinical study, such as hearing or vision loss or a severe cardiac or respiratory disorder. Basic demographic data, motor symptoms, non‐motor symptoms, and HrQoL were assessed and compared with data from 198 patients with idiopathic PD who were matched by age, gender, and disease duration. If the patients were unable to complete the questionnaire, family caregivers were asked to help them. This study’s protocol was approved by the Ethics Committee of Ruijin Hospital affiliated to Shanghai Jiao Tong University School of Medicine. Written informed consent was obtained from all participants prior to the beginning of the study.

### 2.2 Clinical evaluation

Patients completed a questionnaire that inquired about their basic demographic and clinical characteristics, including age, gender, disease duration, educational level, exposure to toxins, family history, and levodopa equivalent daily dose (LEDD). Toxic exposure history was defined as exposure to toxic materials, such as pesticides, herbicides, chemical solvents, and heavy metals for more than 1 year. Patients whose immediate relatives had parkinsonian symptoms were considered to have a family history. We assessed disease severity using the Hoehn and Yahr (HY) staging scale, the presence of pyramidal signs, and the Unified Multiple System Atrophy Rating Scale (UMSARS; Wenning et al., 2004). Non‐motor symptoms were evaluated with a battery of neurological assessment scales: (a) the Mini‐Mental State Examination (MMSE) for cognitive function; (b) the 16‐item Sniffin Sticks odor identification test (SS‐16) for olfaction; (c) the Rapid Eye Movement Sleep Behavior Disorder Screening Questionnaire (RBDSQ) for possible RBD; (d) the Scales for Outcomes in Parkinson’s Disease‐Autonomic questionnaire (SCOPA‐AUT) for autonomic function; (e) the 17‐item Hamilton Rating Scale for Depression (HAMD‐17); and (f) the Hamilton Anxiety Scale (HAMA). Frequencies of cognitive disorders, symptoms of hyposmia, RBD, autonomic dysfunction, depression, and anxiety were calculated using their corresponding cutoff values.

### 2.3 Evaluation of HrQoL

Health‐related quality of life was evaluated using the Medical Outcomes Study 36‐Item Short Form (SF‐36; McHorney, Ware, & Raczek, 1993) and the EuroQol instrument (EQ‐5D; Siderowf & Werner, 2001). The SF‐36 is used worldwide for measurements of HrQoL. It measures eight domains: physical functioning (PF), role physical health (RP), bodily pain (BP), general health perception (GH), vitality (VT), social functioning (SF), role emotion (RE), and mental health (MH). All the domains can be divided into two summary scales: the physical component summary (PCS) and the mental component summary (MCS; McHorney et al., 1993). The EQ‐5D consists of five dimensions: mobility, self‐care, usual activities, pain/discomfort, and anxiety/depression; it has been found to be effective in studies with samples with parkinsonian disorders (Siderowf & Werner, 2001).

### 2.4 Statistical analysis

All continuous demographic and clinical data are presented as mean ± *SD* and analyzed using the *t* test or the Mann–Whitney *U* test. All categorical variables are presented as numbers and analyzed using the chi‐square test. The normality of the variable distributions was evaluated using the Kolmogorov–Smirnov test. An analysis of covariance adjusting for disease duration was conducted to compare differences between the MSA subtypes. Spearman’s correlations were used to detect associations between clinical features and HrQoL. A correlation coefficient above 0.6 was considered strong, between 0.3 and 0.6 was moderate, and below 0.3 was weak or negligible. The multiple linear stepwise regression analysis was performed to explore potential predictors of HrQoL. We used demographic (age, gender, disease duration, and phenotype) and clinical features which were clinically or statistically related with HrQoL (HY scale, UMSARS, HAMD‐17, HAMA, SCOPA‐AUT, and MMSE scores) as independent variables. All tests were 2‐tailed, and the results were considered statistically significant at *p* < 0.05. All statistical analyses were performed using SPSS 20.0 (IBM Corp. Armonk, NY).

## 3 RESULTS

The demographic and clinical characteristics of patients with PD, MSA‐C, and MSA‐P are presented in Table 1. Of the 143 MSA patients, 109 were diagnosed with probable MSA and 34 with possible MSA. A total of 79 patients were male, and 64 were female; their mean age was 61.26 ± 6.93 years; and their mean disease duration was 3.54 ± 1.96 years. Of the 198 patients with PD, 113 were male and 85 were female; their mean age was 61.78 ± 7.65 years; and their mean disease duration was 3.42 ± 1.35 years. They were matched by age, gender, and disease duration when they were recruited for this study. The mean LEDDs were 487.68 ± 383.67 mg/day and 282 ± 254.35 mg/day for the MSA and PD patients, respectively, the mean HY stages were 3.51 ± 1.19 and 1.55 ± 0.55 in the MSA and PD groups, respectively. The frequencies of toxic exposures were 18.9% and 28.3% for the MSA and PD patients, respectively. The frequencies of patients with a family history were 4.2% and 18.2% in the MSA and PD groups, respectively.

**Table 1:** Demographics and clinical characteristics in PD and MSA patients

	MSA *n* = 143	PD *n* = 198	*P* value MSA/PD	MSA‐P *n* = 95	MSA‐C *n* = 48	*P* value^a^ MSA‐P/C	*P* value^b^ MSA‐P/C
Demographic data							
Age	61.26 ± 6.93	61.78 ± 7.65	0.371	61.79 ± 6.80	60.21 ± 7.13	0.199	
Gender (Male/Female)	55.2% (79/64)	57.1% (113/85)	0.737	57.9% (55/40)	50% (24/24)	0.370	
Disease duration	3.54 ± 1.96	3.42 ± 1.35	0.921	3.86 ± 1.91	2.90 ± 1.92	**0.002**	
Education level^c^	33/81/29	32/103/63	**0.039**	23/51/21	10/30/8	0.419	
LEDD (mg/day)	487.68 ± 383.67	282.80 ± 254.35	**0.000**	627.82 ± 367.35	210.31 ± 237.88	**0.000**	**0.000**
Toxic exposure (+/−)	18.9% (27/116)	28.3% (56/142)	0.062	21.1% (20/75)	14.6% (7/41)	0.351	0.390
Family history (+/−)	4.2% (6/137)	18.2% (36/162)	**0.000**	6.3% (6/89)	0% (0/48)	0.179	0.056
Clinical feature							
HY stage	3.51 ± 1.19	1.55 ± 0.55	**0.000**	3.60 ± 1.19	3.33 ± 1.19	0.202	0.819
Pyramid sign (+/−)	51.0% (73/70)	/	/	55.8% (53/42)	41.7% (20/28)	0.111	0.209
OH (+/−)	30.1% (43/100)	/	/	28.4% (27/68)	33.3% (16/32)	0.545	0.526
UMSARS I	19.80 ± 7.97	/	/	21.23 ± 8.23	16.96 ± 6.65	**0.002**	**0.026**
UMSARS II	22.58 ± 10.51	/	/	25.25 ± 9.92	17.29 ± 9.68	**0.000**	**0.000**
UMSARS IV	2.59 ± 1.32	/	/	2.82 ± 1.31	2.13 ± 1.21	**0.002**	**0.019**
RBDSQ	4.07 ± 2.79	3.65 ± 3.27	0.077	3.80 ± 2.88	4.60 ± 2.56	0.094	0.076
RBDSQ (+/−)	46.9% (67/76)	35.9% (71/127)	**0.041**	41.1% (39/56)	58.3% (28/20)	0.051	**0.028**
SS‐16	7.92 ± 0.33	7.58 ± 0.27	0.450	6.94 ± 3.91	8.50 ± 3.79	**0.026**	**0.021**
SS‐16 (+/−)	46.9% (67/76)	48.5% (96/102)	0.766	53.7% (51/44)	33.3% (16/32)	**0.021**	**0.023**
HAMD‐17	9.98 ± 5.19	5.92 ± 4.76	**0.000**	11.10 ± 5.17	7.85 ± 4.58	**0.000**	**0.001**
HAMD‐17 (+/−)	60.1% (86/57)	29.8% (59/139)	**0.000**	67.4% (64/31)	45.8% (22/26)	**0.013**	**0.023**
HAMA	11.01 ± 5.47	7.89 ± 6.30	**0.000**	12.02 ± 5.48	9.19 ± 5.00	**0.004**	**0.013**
HAMA (+/−)	75.5% (108/35)	60.1% (120/78)	**0.004**	77.9% (74/21)	70.8% (34/14)	0.354	0.753
MMSE	25.12 ± 4.57	27.09 ± 3.06	**0.000**	24.58 ± 5.21	26.10 ± 2.85	0.320	**0.044**
MMSE (+/−)	16.1% (23/120)	5.6% (11/187)	**0.001**	22.1% (21/74)	4.2% (2/46)	**0.006**	**0.005**
SCOPA‐AUT	21.26 ± 9.41	10.07 ± 7.93	**0.000**	22.41 ± 9.49	19.02 ± 8.94	**0.042**	0.186

Notes

HAMD‐17: 17‐item Hamilton Rating Scale; HAMA: Hamilton Anxiety Scale; HY stage: Hoehn and Yahr (HY) stage; LEDD: levodopa equivalent daily dosage; MMSE: Mini‐Mental State Examination; MSA: multiple system atrophy; MSA‐C: MSA with prominent cerebellar ataxia; MSA‐P: MSA with predominant parkinsonism; OH: orthostatic hypotension; PD: Parkinson’s disease; RBDSQ: Rapid Eye Movement Sleep Behavior Disorder Screening Questionnaire; SCOPA‐AUT: The Scale for Outcomes in PD autonomic dysfunction; SS‐16: 16‐item odor identification test from Sniffin' Sticks; UMSARS: the Unified Multiple System Atrophy Rating Scale.

a Means the comparison between MSA‐P and MSA‐C.

b Means the comparison between MSA‐P and MSA‐C after adjusting disease duration by analyses of covariance (ANCOVA).

c Education level (primary school and below/middle and high school/bachelor's degree and above). Bold indicates *p* ＜ 0.05.

### 3.1 Comparisons between MSA and PD

No significant differences were found between the PD and MSA patients with respect to age, gender, disease duration, exposure to toxins, RBDSQ scores, or SS‐16 scores. MSA patients had a higher LEDD (*p* < 0.001) and were less likely to have a family history of the disease (*p* < 0.001) than the PD patients. The MSA patients had higher total scores on the HY staging scale (*p* < 0.001), HAMA (*p* < 0.001), HAMD‐17 (*p* < 0.001), and SCOPA‐AUT (*p* < 0.001). The mean score on the MMSE (*p* < 0.001) was lower among the MSA patients than the PD patients.

### 3.2 Comparisons between MSA‐P and MSA‐C

**Figure 1:**
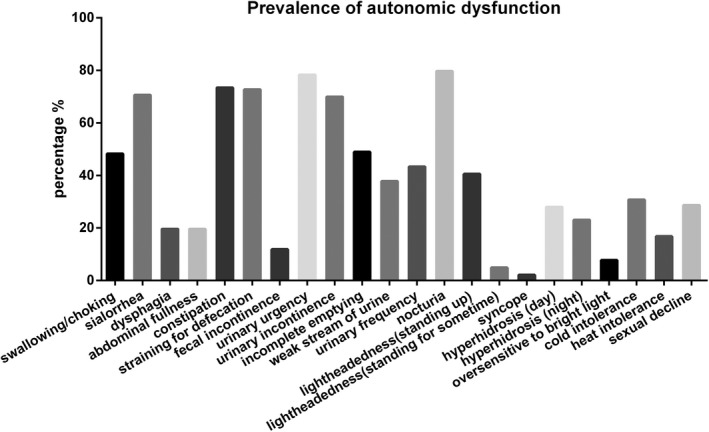
The prevalence of autonomic dysfunction in patients with MSA

Ninety‐five patients (66.4%) were diagnosed with MSA‐P and 48 (33.6%) with MSA‐C. Among the MSA patients, 30.1% had orthostatic hypotension and 51.0% had positive pyramidal signs. A higher LEDD administration (*p* < 0.001) and a longer disease duration (*p* = 0.002) were found in the MSA‐P patients, compared with the MSA‐C patients. The MSA‐P patients had higher scores on the UMSARS I (*p* = 0.026), UMSARS II (*p* < 0.001), UMSARS IV (*p* = 0.019), HAMD‐17 (*p* = 0.001), and HAMA (*p* = 0.013). They had lower scores on the SS‐16 (*p* = 0.021) and the MMSE (*p* = 0.044), compared with MSA‐C patients. The prevalence of symptoms of autonomic dysfunction from high to low was nocturia (79.7%), urinary urgency (78.3%), constipation (73.4%), straining for defecation (72.7%), sialorrhea (70.6%), and urinary incontinence (69.9%; Figure 1). The HrQoL scores on the SF‐36 and EQ‐5D are shown in Table 2. The mean SF‐36 score was 87.71 ± 16.98, and the mean EQ‐5D score was 9.01 ± 1.83. We found that patients with MSA‐P had a lower HrQoL based on their SF‐36 (*p* = 0.006) and EQ‐5D (*p* = 0.037) scores, after adjusting for disease duration.

**Table 2:** Comparison of HrQoL between patients with MSA‐P and MSA‐C

	MSA	MSA‐P	MSA‐C	*P* value	*P*' value^a^
SF‐36 domains					
SF‐36 total	87.71 ± 16.98	84.05 ± 16.33	94.24 ± 16.32	**0.001**	**0.006**
Physical component summary	39.13 ± 15.47	36.58 ± 16.41	44.08 ± 12.06	**0.001**	**0.017**
Mental component summary	39.57 ± 17.20	36.20 ± 17.56	46.23 ± 14.44	**0.000**	**0.001**
Physical function PF	15.41 ± 5.42	15.00 ± 5.05	16.15 ± 6.01	0.516	0.767
Role physical RP	4.13 ± 0.66	4.16 ± 0.76	4.07 ± 0.44	0.442	0.212
Bodily pain BP	9.96 ± 2.73	9.41 ± 2.88	10.85 ± 2.26	**0.002**	**0.015**
General health GH	14.21 ± 2.73	13.80 ± 2.70	14.93 ± 2.65	**0.007**	**0.047**
Vitality VT	14.26 ± 4.50	13.40 ± 4.25	15.78 ± 4.59	**0.004**	**0.007**
Social function SF	5.49 ± 2.22	5.16 ± 2.17	6.09 ± 2.19	**0.017**	0.081
Role emotion RE	4.96 ± 1.34	4.71 ± 1.39	5.41 ± 1.11	**0.004**	**0.006**
Mental health MH	19.49 ± 5.44	18.67 ± 5.26	20.96 ± 5.52	**0.022**	**0.028**
EQ‐5D domains					
EQ‐5D total	9.01 ± 1.83	9.33 ± 1.76	8.43 ± 1.83	**0.002**	**0.037**
Mobility	2.08 ± 0.50	2.09 ± 0.50	2.07 ± 0.49	0.821	0.632
Self‐care	1.95 ± 0.62	2.04 ± 0.62	1.78 ± 0.59	**0.026**	0.076
Usual activities	2.08 ± 0.51	2.10 ± 0.54	2.04 ± 0.47	0.546	0.898
Pain/Discomfort	1.43 ± 0.60	1.52 ± 0.63	1.26 ± 0.49	**0.015**	**0.031**
Anxiety/Depression	1.48 ± 0.532	1.59 ± 0.54	1.28 ± 0.46	**0.002**	**0.002**

EQ‐5D: the EuroQol instrument; HrQoL: health‐related quality of life; MSA: multiple system atrophy; MSA‐C: MSA with prominent cerebellar ataxia; MSA‐P: MSA with predominant parkinsonism; SF‐36: the Medical Outcomes Study 36‐Item Short Form.

a *P*' means the comparison between MSA subtypes after adjusting disease duration by analyses of covariance (ANCOVA). Bold indicates *P* ＜ 0.05.

### 3.3 Correlations between HrQoL and clinical characteristics

Correlations between HrQoL and clinical characteristics are presented in Table 3. The scores on the UMSARS I, UMSARS II, and UMSARS IV correlated strongly with scores on the EQ‐5D (*r* = 0.613, *r* = 0.646, and *r* = 0.646, respectively) and moderately with the scores on the SF‐36 (*r* = −0.575, *r* = −0.579, and *r* = −0.579, respectively). The scores on the UMSARS I, UMSARS II, and UMSARS IV were also correlated with scores on the SF‐36 physical subscale (*r* = −0.506, *r* = −0.490, and *r* = −0.490, respectively), and the SF‐36 mental subscale (*r* = −0.338, *r* = −0.356, and *r* = −0.356, respectively). The HY stage on the HY scale had a weak correlation with the SF‐36 mental summary score (*r* = −0.294) and moderate correlations with scores on the SF‐36 (*r* = −0.569), EQ‐5D (*r* = 0.524), and SF‐36 physical subscale (*r* = −0.468). The HAMD‐17 score had a strong correlation with the SF‐36 score (*r* = −0.650) and moderate correlations with scores on the SF‐36 physical subscale (*r* = −0.331), SF‐36 mental subscale (*r* = −0.525), and the EQ‐5D (*r* = 0.466). Moderate correlations were found between the HAMA score and scores on the SF‐36 (*r* = −0.585), EQ‐5D (*r* = 0.444), SF‐36 physical subscale (*r* = −0.376), and SF‐36 mental subscale (*r* = −0.565). Phenotype had a moderate association with the SF‐36 score (*r* = 0.311) and a weak one with the EQ‐5D score (*r* = −0.279).

**Table 3:** Association between HrQoL and clinical features

	SF‐36		SF‐36 physical summary		SF‐36 mental summary		EQ‐5D	
	*r*	*P* value	*r*	*P* value	*r*	*P* value	*r*	*P* value
Age	−0.089	0.319	−0.157	0.060	0.006	0.948	0.153	0.083
Gender	−0.179	0.043	−0.159	0.058	−0.056	0.508	0.301	0.001
Education	0.084	0.374	0.019	0.836	−0.070	0.435	−0.143	0.128
Phenotype	0.311	0.000	0.270	0.001	0.305	0.000	−0.279	0.001
Duration	−0.259	0.003	−0.269	0.001	−0.141	0.093	0.290	0.001
HY stage	−0.569	0.000	−0.468	0.000	−0.294	0.000	0.524	0.000
UMSARS I	−0.575	0.000	−0.506	0.000	−0.338	0.000	0.613	0.000
UMSARS II	−0.579	0.000	−0.490	0.000	−0.356	0.000	0.646	0.000
UMSARS IV	−0.597	0.000	−0.490	0.000	−0.356	0.000	0.646	0.000
RBDSQ	−0.004	0.962	−0.004	0.961	0.025	0.769	0.034	0.706
SS‐16	0.211	0.018	0.115	0.182	0.126	0.143	−0.173	0.052
HAMD‐17	−0.650	0.000	−0.331	0.000	−0.525	0.000	0.466	0.000
HAMA	−0.585	0.000	−0.376	0.000	−0.565	0.000	0.444	0.000
SCOPA‐AUT	−0.298	0.001	−0.252	0.003	−0.142	0.094	0.263	0.003
MMSE	0.201	0.024	0.209	0.014	0.087	0.311	−0.228	0.010

Note

EQ‐5D: the EuroQol instrument; HAMA: Hamilton Anxiety Scale; HAMD‐17: 17‐item Hamilton Rating Scale; HrQoL: health‐related quality of life; HY stage: Hoehn and Yahr (HY) stage; MMSE: Mini‐Mental State Examination; RBDSQ: Rapid Eye Movement Sleep Behavior Disorder Screening Questionnaire; SCOPA‐AUT: The Scale for Outcomes in PD autonomic dysfunction; SF‐36: the Medical Outcomes Study 36‐Item Short Form; SS‐16: 16‐item odor identification test from Sniffin' Sticks; UMSARS: the Unified Multiple System Atrophy Rating Scale.

The independent variables that were analyzed using linear stepwise regression are shown in Table 4. Depression (HAMD‐17), anxiety (HAMA), global disability (UMSARS IV), and disease severity (HY staging scale) predicted decreased HrQoL, as measured by the SF‐36. Taken together, these variables explained 65.8% (adjusted *R* ^2^) of the variance in SF‐36 scores. Motor and autonomic disability (UMSARS I), depression, and global disability accounted for 53.6% of the variance in EQ‐5D scores. Motor and autonomic disability and anxiety explained 19.9% of the variance in SF‐36 physical subscale scores. Depression and motor impairment explained 32.1% of SF‐36 mental summary scores.

**Table 4:** Determinants of HrQoL in MSA patients

	SF‐36			EQ‐5D			SF‐36 physical subscale			SF‐36 mental subscale		
	*B*	*P*	95% CI	*B*	*P*	95% CI	*B*	*P*	95% CI	*B*	*P*	95% CI
Constant	128.55	0.000	122.41, 134.68	5.25	0.000	4.56, 5.94	56.14	0.000	51.19, 61.08	60.73	0.000	55.67, 65.78
HAMD‐17	−1.35	0.000	−1.85, −0.85	0.09	0.000	0.35, 0.83				−1.25	0.000	−1.63, −0.87
UMSARS IV	−3.38	0.001	−5.32, −1.45	0.59	0.000	0.05, 0.14				−2.01	0.009	−3.52, −0.51
HY stage	−3.65	0.001	−5.69, −1.60									
HAMA	−0.54	0.025	−0.99, −0.07				−0.323	0.044	−0.64, −0.01			
UMSARS I				0.067	0.002	0.024, 0.109	−0.515	0.000	−0.75, −0.28			
Adjusted *R* ^2^	0.658			0.536			0.199			0.321		

Note

B: regression coefficient; CI: confidence interval; EQ‐5D: the EuroQol instrument; HAMA: Hamilton Anxiety Scale; HAMD‐17: 17‐item Hamilton Rating Scale; HrQoL: health‐related quality of life; HY stage: Hoehn and Yahr stage; MSA: multiple system atrophy; SF‐36: the Medical Outcomes Study 36‐Item Short Form; UMSARS: the Unified Multiple System Atrophy Rating Scale.

## 4 DISCUSSION

This investigation was a comprehensive large‐scale study conducted with a Chinese sample. Although PD and MSA are parkinsonian syndromes and have similar symptoms, such as emotional changes and autonomic problems, patients with MSA performed significantly worse on measures of motor impairment, depression, anxiety, autonomic dysfunction, and cognitive impairment compared with PD patients. No significant difference was found in RBD scores; however, the frequency of RBD was higher in the MSA (46.9%) than the PD patients (35.9%), which is consistent with one study (Ghorayeb et al., 2002), but lower than another study (Palma et al., 2015) showing that approximately 73% of the patients with MSA had suspected RBD. Different sample sizes and populations might explain the different results. Patients with MSA sometimes report a gradual disappearance of RBD syndrome, as the disease progresses. In a previous study (Nomura et al., 2011), the frequency of RBD increased with the progression of the disease in PD patients, but gradually decreased after the onset of MSA. However, the same finding was not observed in our study, and research with larger sample needs to be conducted. There were no significant differences in olfaction between MSA and PD groups in our study. Several studies have supported the values of olfactory testing in distinguishing MSA and PD (Fujita et al., 2016; Izawa et al., 2012). They found that olfactory loss was more frequently observed in PD patients. In studies on the differentiation of PD from MSA, olfactory loss was the result of alpha‐synuclein aggregation in the olfactory bulb and olfactory tract in PD patients (Chen et al., 2014). We found a lower SS‐16 score only among the PD patients. The poor mental state and attention of the MSA patients might have had negative effects on the olfactory testing.

The predominant parkinsonian features were detected in 95 (66.4%) MSA patients in our study, which was different from a study that reported the cerebellar subtype was the main phenotype among Japanese patients (Ozawa & Onodera, 2017). After adjusting for disease duration, the MSA‐P patients tended to have higher USMARS scores, which suggest more severe motor impairment and disability compared with the MSA‐C patients. One study reported that parkinsonian features were later observed in some patients who were initially diagnosed with MSA‐C, while changes in the predominant features were not observed in MSA‐P patients (Yabe et al., 2006). It seemed that the progression of parkinsonism was more rapid and severe than cerebellar ataxia among the MSA patients. Because it is easy to misdiagnose MSA as PD and the levodopa‐response of the patients was poor in the early stage of the disease, the patients may have reported a longer duration of the disease and a larger LEDD by the time they were diagnosed with MSA‐P. We also found that severe depression, anxiety, hyposmia, and cognitive deficits were more common among MSA‐P than MSA‐C patients. Anxiety (75.5%) and depression (60%) were common symptoms among MSA patients in this study, which is consistent with the findings of a previous study (Zhang et al., 2018). Regarding the mechanisms of depression and anxiety in MSA, research on cerebral glucose metabolism has revealed that dorsolateral prefrontal dysfunction contributes to depression in MSA (Herting et al., 2007). In our study, 15.1% of the MSA patients showed cognitive deficits on the MMSE, which was also found in another study that used the MMSE (Cao et al., 2015). In the second version of the MSA diagnostic criteria, cognitive deficit was not included as a backup feature (Gilman et al., 2008). However, recent studies have found that cognitive deficits in MSA may be underestimated (Cao et al., 2015; Koga et al., 2017). Approximately 32% of MSA patients have cognitive deficits, and frontal‐executive disturbance is the most common vulnerable domain (Koga et al., 2017). In our study, MSA‐P patients presented with worse cognitive function than MSA‐C patients, which is consistent with previous research indicating that the cognitive dysfunction in MSA‐P patients may be the result of prefrontal involvement (Kawai et al., 2008). Given the limitations of the MMSE (Scazufca, Almeida, Vallada, Tasse, & Menezes, 2009), a detailed analysis of cognitive domains between the different groups was not conducted.

Autonomic dysfunction, a central feature of MSA, was evaluated using the SCOPA‐AUT in our study. Almost all of the MSA patients suffered from autonomic failure, which included bladder, bowel, and sexual disturbances, orthostatic hypotension, and abnormal sweating. Urinary dysfunction was the most common autonomic symptom in our study. A retrospective study found that urinary dysfunction was reported by 96% of patients and may precede motor symptoms by several years (Kirchhof et al., 2003). In this study, MSA patients had worse autonomic performance than the PD patients, and previous studies have indicated that autonomic function tests can play a role in the differential diagnosis between MSA and PD (Kimpinski et al., 2012). Autonomic failure in MSA is caused by preganglionic neuronal loss and α‐synuclein‐positive glial and neuronal cytoplasmic inclusions in the medullar and spinal autonomic nuclei, whereas autonomic pathology in PD is postganglionic (Braak, de Vos, Bohl, & Del Tredici, 2006).

The comparison of the SF‐36 and EQ‐5D between the two subtypes, after adjusting for disease duration, showed that MSA‐P patients had worse PCS and MCS, as indicated by their scores on the SF‐36, and worse pain/discomfort and anxiety/depression, according to their scores on the EQ‐5D. The presence of parkinsonism seemed to bring about a more serious decline in HrQoL than the presence of cerebellar dysfunction, which is inconsistent with the results of another study (Winter et al., 2011). HrQoL was negatively associated with increased motor impairment and disease severity, as assessed by the HY staging scale and the UMSARS, indicating that disease progression contributed to the deterioration of HrQoL, previous studies have also confirmed these results (Chen et al., 2014; Schrag et al., 2006, 2010; Winter et al., 2011). Depression and anxiety scores were moderately correlated with HrQoL scores; other studies have reported a close association between depression and HrQoL (Schrag et al., 2006, 2010; Winter et al., 2011; Zhang et al., 2017). Autonomic dysfunction was correlated with HrQoL in one study (Schrag et al., 2006), but only a weak correlation with the HrQoL was found in the present study. Finally, the multiple linear stepwise regression analysis showed that depression, anxiety, degree of disability, and disease severity predicted decreased HrQoL. One study reported that autonomic dysfunction, depression, and motor impairment were closely associated with poor HrQoL among MSA patients (Schrag et al., 2006). Another investigation with MSA patients found that depression, anxiety, and motor impairment were independent predictors of HrQoL (Schrag et al., 2010). Given the limitations of treatments for MSA, more attention should be paid to improving patients’ HrQoL by providing symptomatic treatment for depression and anxiety, which might involve patients’ families, the healthcare system, and support for motor impairment.

This study has some limitations. First, we used a cross‐sectional design; therefore, a longitudinal evaluation of symptoms was not conducted. In our research, parkinsonism progressed faster and was more severe than cerebellar ataxia in the MSA patients; however, this needs to be verified in future studies. Second, the sample size should be increased to avoid selection bias. Third, some patients are in the early stages of the disease and have not yet fully met the diagnostic criteria for probable MSA. MSA is not a very common disease, it is not easy for all patients to meet the probable diagnosis, and future studies should select probable MSA patients exclusively.

## 5 CONCLUSIONS

In conclusion, motor impairment, depression, anxiety, autonomic dysfunction, and cognitive impairment seemed to be much more serious in patients with MSA than those with PD. Our findings indicate that patients with MSA‐P have more severe symptoms of motor impairment, hyposmia, depression, anxiety, and cognitive impairment than those with MSA‐C. Our results also suggest that MSA‐P patients also have a lower HrQol with respect to their physical and mental health. The independent predictors of poor HrQoL were depression, anxiety, the degree of disability, and disease severity. Therefore, more attention should be paid to improving patients’ HrQoL considering the limitations of treatments for MSA.

## CONFLICT OF INTEREST

None declared.

## AUTHORS CONTRIBUTION

Shengdi Chen helped designing the study. The study was organized by Juanjuan Du. The data collection works were completed by the study group including Juanjuan Du, Tian Wang, Pei Huang, Shishuang Cui, Chao Gao, Yiqi Lin, Rao Fu. Finally, Juanjuan Du finished the statistical analysis and article writing. Sheng‐di Chen supervised the study and revised the manuscript.
